# Evaluation of the antimicrobial potential of fluoride-free mouthwashes against *Scardovia wiggsiae*

**DOI:** 10.1007/s40368-025-01116-4

**Published:** 2025-10-08

**Authors:** A. C. Valdivia-Tapia, F. Lippert, R. L. Gregory

**Affiliations:** 1 Indiana University, School of Dentistry, Department of Biomedical and Applied Sciences, Indianapolis, USA; 2 Indiana University, School of Dentistry, Oral Health Research Institute, Indianapolis, USA; 3 Indiana University, School of Medicine, Pathology Laboratory Medicine, Indianapolis, USA

**Keywords:** *Scardovia wiggsiae*, Fluoride-free mouthwash, Antimicrobial efficacy, Biofilm inhibition

## Abstract

**Purpose:**

This study aimed to evaluate the antimicrobial efficacy of fluoride-free mouthwashes against *Scardovia wiggsiae*, a key bacterium associated with fluoride-resistant dental caries. The investigation focused on bacterial growth inhibition, planktonic cell viability, and biofilm formation.

**Methods:**

Twelve commercially available fluoride-free mouthwashes with various active ingredients, including hydrogen peroxide, cetylpyridinium chloride (CPC), essential oils, and organic extracts, were tested. Minimum inhibitory concentration (MIC) and minimum bactericidal concentration (MBC) assays were conducted to determine bacterial growth inhibition and killing potential. Planktonic cell viability and biofilm formation assays were performed at three dilutions (1:3, 1:6, and 1:12) using a 16-h culture of *S. wiggsiae*. Optical density (OD) values were measured at 595 nm for planktonic cells and 490 nm for biofilms.

**Results:**

Significant variability was observed in bacterial inhibition among mouthwashes. Hydrogen peroxide- and CPC-based formulations demonstrated the strongest antimicrobial activity, significantly reducing bacterial growth, planktonic viability, and biofilm formation. Essential oil-based formulations exhibited moderate antimicrobial effects, with reduced efficacy at higher dilutions. Organic-based mouthwashes showed limited inhibition, while formulations containing zinc chloride and stabilised chlorine dioxide demonstrated the weakest effects.

**Conclusion:**

Hydrogen peroxide- and CPC-containing mouthwashes exhibited the highest antimicrobial potential against *S. wiggsiae* and may serve as effective fluoride-free alternatives for high-risk populations. Essential oil-based formulations provided moderate benefits, whereas zinc chloride and chlorine dioxide showed minimal efficacy. These findings underscore the importance of selecting appropriate antimicrobial agents for biofilm control and caries prevention. Further in vivo studies are necessary to validate long-term effectiveness in clinical settings.

## Introduction

Oral health is a vital component of overall well-being, with microbial biofilms playing a central role in the development of dental diseases such as dental caries and periodontal disease (Bertolini et al. 2022). While fluoride-based interventions have long been the cornerstone of caries prevention (Pitts et al. 2017; Clark et al 2020; Toumba et al. 2019), concerns about fluoride resistance (Cai et al. 2017; Sun et al. 2024) limited efficacy in high-risk populations, and potential adverse effects have prompted interest in alternative antimicrobial agents (Brookes et al. 2023; Valdivia-Tapia et al. 2025a, b). Among the emerging bacterial species associated with oral dysbiosis, *Scardovia wiggsiae* has garnered attention, due to frequent detection in caries that progress despite routine fluoride exposure and in biofilms that show reduced susceptibility to fluoride patterns observed particularly in paediatric and other high-risk populations (Kressirer et al. 2017; Kameda et al. 2020).

As a facultative anaerobe, *S. wiggsiae* thrives in acidic, low-pH environments and often coexists with other cariogenic species such as *Streptococcus mutans* and *Lactobacillus spp*., contributing to highly acidogenic and fluoride-resistant biofilms (Kameda et al. 2020). The persistence *of S. wiggsiae* despite fluoride exposure presents a unique clinical challenge, emphasising the need for alternative strategies to disrupt cariogenic biofilms.

Fluoride-free mouthwashes have emerged as potential adjuncts for biofilm control, utilising active ingredients such as hydrogen peroxide, cetylpyridinium chloride (CPC), essential oils, and stabilised chlorine dioxide, which exhibit antimicrobial properties without relying on fluoride (Takenaka et al. 2022; Hossainian et al. 2011; Paraskevas et al. 2008; Dong et al. 2012). Several studies have demonstrated that such agents can reduce overall bacterial load, inhibit plaque accumulation, and lower gingival inflammation, supporting their role in improving oral health (Takenaka et al.,2022; James et al., 2017). Importantly, by targeting key microbial components of cariogenic biofilms, these formulations may contribute to caries prevention, particularly when fluoride exposure is insufficient. It is important to note that there is no evidence that the use of antimicrobial mouthwashes has a positive effect on decreasing caries risk.

However, despite the growing clinical use of fluoride-free formulations (Valdivia Tapia et al., submitted, 2025 Valdivia-Tapia et al. 2025b), their specific effects on *S. wiggsiae* remain largely unexplored. As *S. wiggsiae* has been increasingly linked to high caries risk, therefore understanding whether fluoride-free mouthwashes can inhibit its growth and biofilm formation is crucial for informing evidence-based clinical recommendations.

The present study aims to evaluate the antimicrobial potential of various fluoride-free mouthwashes against *S. wiggsiae*, with a focus on their ability to reduce total bacterial growth, inhibit planktonic cell formation, and disrupt biofilm development.

## Materials and methods

The present study followed an in vitro experimental design to evaluate the antimicrobial activity of fluoride-free mouthwashes against *S. wiggsiae* using MIC, MBC, planktonic cell viability, and biofilm inhibition assays. A total of twelve commercially available mouthwashes with various active ingredients were tested. Three dilutions (1:3, 1:6, and 1:12) were prepared for each mouthwash using tryptic soy broth supplemented with 1% sucrose (TSBS). A 16-h culture of *S. wiggsiae* strain F_0424_ (culture kindly provided by Dr. Anne Tanner, Forsyth Institute, Boston, MA) was used as the bacterial inoculum. The antimicrobial efficacy of the mouthwashes was assessed using multiple assays: MIC (minimum inhibitory concentration) to determine bacterial growth inhibition, MBC (minimum bactericidal concentration) to assess bacterial killing potential, planktonic cell viability to measure the effect on free-floating bacterial cells, and biofilm formation to evaluate the impact on adherent bacterial communities. A negative control (TSBS only) to establish baseline bacterial growth was selected. The optical density (OD) values were measured at 595 nm for MIC and planktonic cell viability assays, while 490 nm was used for biofilm quantification (Batra et al. 2022). The data were statistically analysed to determine the relative efficacy of each mouthwash. This experimental approach provided a comprehensive evaluation of different fluoride-free mouthwash formulations and their potential antimicrobial effects against *S. wiggsiae.*

### Preparation of the samples

Twelve different mouthwashes were purchased in Indianapolis, Indiana, USA. Three different serial dilutions (1:3, 1:6, and 1:12) in TSBS were prepared. Two controls were prepared: The negative control was only with TSBS, and the positive control was 0.12% chlorhexidine (chlorhexidine digluconate solution, 20% water, Sigma) prepared in TSBS (Table 1).

**Table 1:** Treatments

Code	Commercial name	Active ingredient
A	Colgate optic white high-impact white advanced	Hydrogen peroxide
B	Oral B gum detoxify	Cetylpyridinium chloride 0.07%
C	Listerine freshburst	Eucaliptol (0.092%), menthol (0.042%), methyl salicylate (0.060%) thymol (0.064%), Alcohol. 21.6%
D	Listerine ultra clean zero alcohol	Eucaliptol (0.092%), menthol (0.042%), methyl salicylate (0.060%) thymol (0.064%)
E	Crest pro-health intense	Cetylpyridinium chloride 0.05%, alcohol 15%
F	Jason healthy mouth tartar control cinnamon clove	Organic ingredient (Cinnamon)
G	Parodontax active gum health mint	Cetylpyridinium chloride 0.07%
H	Crest bacteria blast	Sodium hexametaphosphate, hydrogen peroxide, alcohol 5%
I	CloSYS	Stabilised chloride dioxide
J	Listerine sensitivity zero alcohol	Dipotassium oxalate monohydrate, sodium laurylsulfate
K	Smart mouth original	Zinc chloride
L	Smart mouth mouth sore zinc-activated oral rinse	Menthol 0.2% (when mixed directed), zinc chloride

#### Preparation of the samples

Twelve different mouthwashes were purchased from commercial sources in Indianapolis, Indiana, USA. To assess their antimicrobial potential, three serial dilutions were prepared for each mouthwash: 1:3, 1:6, and 1:12, using TSBS as the diluent. Two control groups were included in the study: negative control (TSBS alone), with no antimicrobial agents; and positive control: 0.12% chlorhexidine digluconate solution (Sigma-Aldrich, St. Louis, MO, USA) diluted in TSBS, a known standard antimicrobial agent for comparison. The diluted mouthwash samples were used in subsequent microbiological assays to evaluate their efficacy against *S. wiggsiae.*

#### Minimum inhibitory concentration (MIC), minimum bactericidal concentration (MBC), planktonic cell viability, and biofilm formation assays

A 16-h culture of *S. wiggsiae* was grown in TSBS at 37 °C under 5% CO₂ conditions to prepare the bacterial inoculum. All experiments were performed in quadruplicate and repeated three independent times to ensure reproducibility. To assess the minimum inhibitory concentration (MIC) of each mouthwash, 10 µL of the 16-h bacterial culture was inoculated into 190 µL of each prepared mouthwash dilution in a sterile 96-well flat-bottom microtiter plate (Fisher Scientific, Newark, DE, USA). The plates were incubated at 37 °C under 5% CO₂ for 24 h. The optical density (OD) of the bacterial cultures was measured at 595 nm using a spectrophotometer (SpectraMax 190; Molecular Devices, Sunnyvale, CA, USA). The MIC was determined as the lowest concentration at which a clear-cut reduction in absorbance was observed, indicating significant bacterial inhibition of growth.

Following MIC determination, 5 µL from each well of the microtiter plate was spotted onto fresh blood agar plates and incubated for 48 h at 37 °C under 5% CO₂. The minimum bactericidal concentration (MBC) was defined as the lowest concentration of mouthwash that resulted in no visible bacterial colony growth on the blood agar plate.

To assess the effect of mouthwashes on planktonic cell viability, 120 µL of the unbound planktonic cell suspension was aspirated from each well and transferred to a new 96-well plate. The OD at 595 nm was measured to quantify the remaining planktonic bacterial growth.

To evaluate the ability of mouthwashes to inhibit biofilm formation, the remaining planktonic cells were removed from the original biofilm microtiter plate, leaving only attached biofilm-forming bacteria. The wells were treated as follows: biofilm fixation: 200 µL of 10% formaldehyde was added to each well and incubated for 30 min to fix the biofilm. Biofilm staining: formaldehyde was removed, and the wells were washed three times with deionised water. Then, 200 µL of 0.5% crystal violet dye was added, and the cells were stained for 30 min. Stain extraction: the wells were rinsed three times, and 200 µL of 2-isopropanol was added for one hour to lyse the biofilm cells and extract the crystal violet dye. Biofilm quantification: the OD was measured at 490 nm using a spectrophotometer to determine the extent of biofilm formation.

## Results

Table 2 presents the *p* values obtained from a two-way ANOVA evaluating the effects of *Treatment*, *Dilution*, and their interaction (*Treatment* × *Dilution*) on three parameters: total absorbance, planktonic, and biofilm bacterial populations. Statistically significant results (*p* < 0.05) indicate that both the individual factors and their interaction significantly influence the outcomes. This means the effectiveness of the treatments depends on the dilution level as some mouthwashes are only effective at certain concentrations.

**Table 2:** Two-Way ANOVA p values for the effects of treatment and dilution on bacterial populations

	*p* value		
	Total absorbance	Planktonic	Biofilm
Treatment	< 0.001	< 0.001	0.006
Dilution	< 0.001	< 0.001	< 0.001
Treatment * Dilution	< 0.001	< 0.001	< 0.001
Alfa = 0.05			

Table 3 shows the p values from post hoc pairwise comparisons between different treatment dilutions (1:3, 1:6, and 1:12) for total absorbance, planktonic, and biofilm bacterial populations. The analysis was conducted to assess the impact of dilution on antimicrobial effectiveness. Statistically significant differences (*p* < 0.05) indicate that the antimicrobial effects varied significantly between the tested dilutions. Significant differences were found between 1:3 vs 1:6, and 1:3 vs 1:12 across all parameters. However, 1:6 vs 1:12 showed no significant difference for biofilm formation (*p* = 0.327), suggesting that increasing dilution beyond 1:6 does not significantly reduce biofilm effectiveness in some cases. Overall, more concentrated dilutions (1:3) tend to be more effective, especially in reducing biofilm.

**Table 3:** Pairwise comparisons of antimicrobial efficacy between different dilutions

Dilution	Comparison		*p* value		
			Total absorbance	Planktonic	Biofilm
1:3		1:6	0.017	0.001	0.002
1:3		1:12	< 0.001	< 0.001	0.000
1:6		1:12	< 0.001	< 0.001	0.327

The antimicrobial activity of various fluoride-free mouthwashes against *S. wiggsiae* was assessed through measurements of total absorbance, planktonic and biofilm bacterial absorbance, as well as minimum bactericidal concentration (MBC) values (Table 4). Treatments A and H demonstrated the greatest efficacy, with significantly reduced total absorbance and complete elimination of planktonic cells. Notably, both exhibited MBC values greater than 1:12, the same as chlorhexidine (> 1:12), indicating potent bactericidal activity even at high dilutions. Treatment A showed minimal biofilm formation, while H, although effective against planktonic bacteria, presented slightly elevated biofilm levels compared to other high-performing agents.

**Table 4:** Antimicrobial efficacy of various fluoride-free mouthwashes against *S. wiggsiae,* measured by total absorbance, planktonic, and biofilm bacterial absorbance

	Total absorbance*	Planktonic*	Biofilm*	MBC**
Negative control	0.453 ± 0.020 g	0.010 ± 0.000^abc^	0.000 ± 0.000^a^	< 1:3
A	0.014 ± 0.020 ab	0.000 ± 0.000^a^	0.001 ± 0.000^a^	> 1:12
B	0.278 ± 0.089 e	0.100 ± 0.016^f^	0.003 ± 0.048^a^	> 1:12
C	0.261 ± 0.155 e	0.009 ± 0.070^abc^	0.004 ± 0.004^a^	< 1:3
D	0.126 ± 0.085 cd	0.037 ± 0.043^de^	0.006 ± 0.005^a^	< 1:3
E	0.121 ± 0.041 cd	0.022 ± 0.012^cd^	0.006 ± 0.045^a^	> 1:12
F	0.383 ± 0.125 f	0.053 ± 0.013^e^	0.016 ± 0.062^a^	< 1:3
G	0.110 ± 0.180 cd	0.024 ± 0.042^cd^	0.016 ± 0.017^a^	< 1:12
H	0.006 ± 0.010 a	0.000 ± 0.000^a^	0.023 ± 0.001^a^	> 1:12
I	0.296 ± 0.272 e	0.003 ± 0.006^ab^	0.064 ± 0.009^b^	< 1:12
J	0.160 ± 0.232 d	0.021 ± 0.034^bcd^	0.082 ± 0.020^bc^	< 1:12
K	0.094 ± 0.144 cd	0.001 ± 0.003^a^	0.090 ± 0.006^c^	< 1:12
L	0.077 ± 0.159 bc	0.000 ± 0.000^a^	0.114 0.001^d^	< 1:12

Results are expressed as mean ± standard deviation. The minimum bactericidal concentration (MBC) was also expressed

*Lowercase letters denote statistical groupings; values sharing the same letters are not significantly different (*p* > 0.05)

**MBC values represent the lowest dilution at which no bacterial growth was observed. *MBC values* > *1:12* indicate that the mouthwash was bactericidal at high dilution. *MBC values* < *1:3* indicate a lower bactericidal effect, requiring a more concentrated formulation for bacterial inhibition

These different letters indicate that values sharing the same letters are not significantly different (*p* 0.05)

Moderately effective treatments included C, D, E, and G, which generally demonstrated reduced total absorbance and moderate control of planktonic and biofilm populations. However, some (C and D) required more concentrated formulations, as reflected by their MBC values below 1:3. On the other hand, treatments such as B and F had relatively high absorbance and planktonic values, despite MBCs above 1:12, suggesting that while bactericidal activity may occur at lower concentrations, these formulations are less efficient in reducing the overall bacterial mass.

Several treatments, including I, J, K, and L, were associated with higher levels of biofilm formation. Although some of these had low planktonic counts or total absorbance, their inability to effectively inhibit biofilm formation limits their potential clinical utility against *S. wiggsiae*. The control group exhibited the highest total absorbance, validating the antimicrobial effects of the test agents. Statistical grouping indicated that many of the most effective treatments shared overlapping significance markers, especially in planktonic reduction, but differed in their impact on biofilm.

**Fig. 1:**
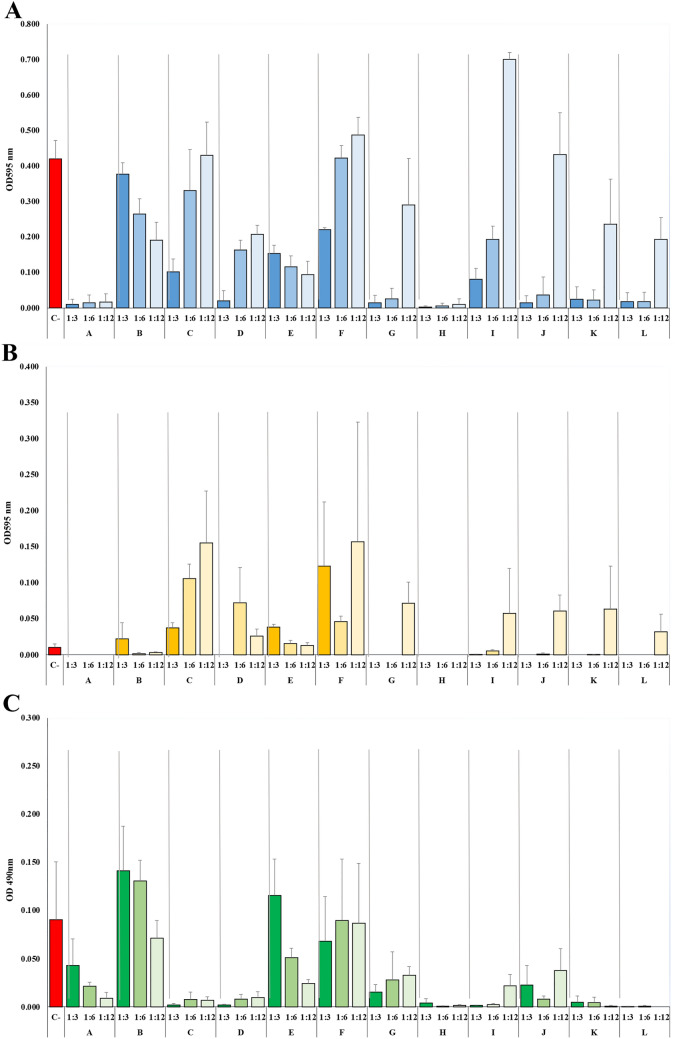
**A** Effect of different mouthwashes with different active ingredients on total absorbance formation of *S. wiggsiae* (3 dilutions: 1:3, 1:6, 1:12; three different times). **B** Effect of different mouthwashes with different active ingredients on planktonic cell formation of *S. wiggsiae* (3 dilutions: 1:3, 1:6, 1:12; three different times). **C** Effect of different mouthwashes with different active ingredients on biofilm formation of *S. wiggsiae* (3 dilutions: 1:3, 1:6, 1:12; three different times)

The effect of different mouthwashes on *S. wiggsiae* total absorbance at three dilution levels (1:3, 1:6, and 1:12) was measured by absorbance at 595 nm without shaking (Fig. 1A). Results reveal variability in bacterial inhibition among formulations. The negative control (C−) exhibited the highest absorbance, indicating strong bacterial growth. Mouthwashes such as C, D, and F showed low absorbance across all dilutions, suggesting strong inhibition, while I and J have higher absorbance at 1:12, indicating reduced efficacy with dilution. Acidic mouthwashes (CPC and EO-based) indicate decreasing inhibition as dilution increases, likely due to reduced antimicrobial potency. Conversely, some fluoride and herbal-based mouthwashes maintained their effects even at lower concentrations. These findings highlight the impact of active ingredients, pH, and dilution on antibacterial efficacy.

The effect of different mouthwashes on planktonic cell formation of *S. wiggsiae* at three dilution levels (1:3, 1:6, and 1:12) was measured by absorbance at 595 nm with shaking (Fig. 1B). The negative control (C−) exhibited the highest absorbance, indicating strong bacterial growth. Mouthwashes C, D, and F showed higher absorbance, suggesting weaker inhibition, especially at 1:12, while A, B, and H displayed low absorbance, indicating stronger antimicrobial activity. Acidic formulations (CPC and EO-based) showed reduced inhibition with increasing dilution, whereas some fluoride and herbal-based mouthwashes maintained their effects across concentrations. These findings highlight the impact of active ingredients, pH, and dilution on planktonic bacterial growth.

The effect of different mouthwashes on biofilm formation of *S. wiggsiae* at three dilution levels (1:3, 1:6, and 1:12) was measured by absorbance at 490 nm (Fig. 1C). The negative control (C−) showed the highest absorbance, indicating strong biofilm formation. Mouthwashes F and G exhibited higher absorbance values, suggesting weaker inhibition, particularly at 1:12. In contrast, A, B, H and J displayed lower absorbance, indicating stronger antimicrobial activity against biofilm formation. Acidic mouthwashes (CPC and EO-based) showed reduced biofilm inhibition with increasing dilution, while some fluoride and herbal-based formulations maintained their effects across concentrations. These findings underscore the role of active ingredients, pH, and dilution in controlling biofilm growth.

## Discussion

The primary aim of the present study was to evaluate the antimicrobial potential of fluoride-free mouthwashes against *S. wiggsiae*, a bacterium increasingly associated with fluoride-resistant dental caries. Given its ability to thrive in acidic and fluoride-rich environments, *S. wiggsiae* poses a unique challenge in caries management (Kameda et al. 2020). This study focused on assessing the impact of various mouthwashes on bacterial growth, planktonic cell viability, and biofilm formation to identify effective alternative antimicrobial agents for oral health maintenance.

Our findings indicate that mouthwashes containing hydrogen peroxide (A, H) and cetylpyridinium chloride (B, E, G) exhibited the most potent antimicrobial effects against *S. wiggsiae*. These formulations significantly reduced bacterial growth, planktonic cell viability, and biofilm formation, suggesting strong bactericidal and anti-biofilm properties. Hydrogen peroxide exerts its effect through oxidative stress, leading to bacterial membrane damage and cell death (Hossainian et al. 2011). Cetylpyridinium chloride, a quaternary ammonium compound, is known to disrupt bacterial adhesion, reducing biofilm formation, which aligns with previous studies demonstrating its effectiveness against oral pathogens (Haps et al. 2008; Versteeg et al. 2010; Pitten and Kramer 2001).

Mouthwashes containing essential oils (C, D) showed a concentration-dependent antimicrobial effect, with stronger inhibition at lower dilutions (1:3). Essential oils such as eucalyptol, thymol, and menthol disrupt bacterial cell membranes and interfere with quorum sensing, which is crucial for biofilm development (Stoeken et al. 2007). However, their effectiveness was reduced at higher dilutions, suggesting that repeated or prolonged exposure may be necessary for sustained bacterial inhibition. These findings support previous research demonstrating the antimicrobial properties of essential oils but highlight the need for optimisation in formulation and concentration.

Organic-based formulations, particularly mouthwash F (cinnamon and clove extract), exhibited moderate antibacterial effects. These plant-derived compounds possess natural antimicrobial and anti-inflammatory properties (Ranasinghe et al. 2013); however, their variability in effectiveness suggests that they may require higher concentrations or complementary antimicrobial agents to enhance their efficacy. Prior studies have shown that plant-based antimicrobials can reduce bacterial viability, but their long-term effects on cariogenic biofilms remain uncertain (Alshahrani and Gregory 2020).

In contrast, mouthwashes containing zinc chloride (K, L) and stabilised chlorine dioxide (I) showed relatively weaker antimicrobial activity. Zinc chloride is known to have anti-plaque properties, but its primary mechanism involves protein precipitation rather than direct bacterial inhibition (Lynch 2011). Stabilised chlorine dioxide, on the other hand, functions primarily as an oxidising and deodorising agent rather than a potent antimicrobial; however, a recent report indicated that it kills other oral microbes (Ayoub et al. 2023).

In interpreting the findings, it is important to note that chlorhexidine (CHX) can precipitate proteins on contact with bacterial and matrix components, increasing turbidity and absorbance in MIC/biofilm assays and potentially mimicking growth; in our study, subculture-based MBC testing (> 1:12) showed no viable colonies at any CHX dilution, indicating that the elevated absorbance reflected precipitation rather than survival. Beyond simple persistence in acidic plaque, *S. wiggsiae* appears to contribute actively to cariogenicity: It is strongly acidogenic, producing acetate and lactate via the fructose-6-phosphate phosphoketolase pathway, and remains metabolically active at low pH (aciduric), often coexisting with *S. mutans* in high-risk settings such as severe early childhood caries, orthodontic plaque, and root-caries biofilms (Kressirer et al. 2017; Tanner et al. 2011; Kameda et al. 2020; Damé-Teixeira et al., 2020).

Within this context, the superior in vitro performance of fluoride-free formulations containing hydrogen peroxide or cetylpyridinium chloride suggests potential adjunctive roles for patients at elevated caries risk who prefer fluoride-free options, whereas essential-oil and organic formulations showed more modest effects and zinc chloride or stabilised chlorine dioxide may require reformulation or combination therapy to enhance activity against *S. wiggsiae*. These observations align with prior reports on the acidogenic, aciduric ecology of cariogenic biofilms and support further work in dynamic, clinically reflective models and longitudinal trials to determine whether the observed antimicrobial effects translate into meaningful caries-risk reduction.

Previous research has highlighted the role of *S. wiggsiae* in fluoride-resistant caries and its association with polymicrobial biofilms in dental lesions. Tanner et al. (2018) identified *S. wiggsiae* as a key pathogen in severe early childhood caries, frequently detected alongside *S. mutans*. Kameda et al. (2020) demonstrated that *S. wiggsiae* thrives at low pH and persists under fluoride exposure. Mechanistically, *S. wiggsiae* contributes to acidogenic biofilms by (i) fermenting dietary carbohydrates via the fructose-6-phosphate phosphoketolase pathway to produce high levels of organic acids (predominantly acetate and lactate) (Ruas-Madiedo et al. 2005; Sánchez et al. 2005; Kameda et al., 2020; Manome et al., 2019), (ii) tolerating and continuing metabolism at low pH (aciduricity), thereby sustaining sub-critical pH within the biofilm (Valdez et al. 2016) and (iii) coexisting synergistically with *S. mutans* and other cariogenic taxa, which enhances matrix accumulation and acid persistence (Tanner et al. 2011; Kressirer et al. 2017; Kameda et al. 2020). The present findings align with these studies by confirming *S. wiggsiae* ability to form biofilms and resist conventional antimicrobial agents, underscoring the importance of identifying effective fluoride-free alternatives.

In addition to conventional antimicrobial agents, increasing attention has been directed towards non-toxic and biocompatible substitutes for the management of caries and gingivitis. Recent studies highlight that plant-derived polyphenols and natural extracts exhibit both antibacterial and anti-inflammatory properties, making them promising adjuncts in oral care. For example, catechins and flavonoids have demonstrated inhibitory effects on cariogenic bacteria and suppression of inflammatory mediators in gingival tissues (Ripari et al. 2020a). Similarly, polyphenol-rich formulations derived from green tea and cranberries have been shown to reduce plaque accumulation, limit biofilm formation, and modulate host immune responses without the cytotoxic effects often associated with synthetic antimicrobials (Ripari et al. 2020b) More recently, innovative biomaterials enriched with natural compounds have shown potential not only in controlling cariogenic biofilms but also in attenuating gingival inflammation, further supporting their clinical applicability as safe alternatives to traditional chemical agents (Zumbo et al. 2023). These findings collectively suggest that the incorporation of non-toxic substitutes into oral hygiene products could provide an effective dual action bacterial inhibition and inflammation control while improving tolerability and patient acceptance.

Despite the valuable insights gained, the present study had limitations that should be considered. The study was conducted under laboratory conditions, which do not fully replicate the oral environment’s complexities, such as salivary flow, dietary influences, and host immune responses. The antimicrobial effects were assessed over a limited period; the long-term effects of repeated mouthwash use were not evaluated. While *S. wiggsiae* is relevant in caries lesion formation it is enriched in cavitated severe early childhood caries lesions, produces high levels of acetate/lactate via the F6PPK (bifid) pathway, grows and metabolises at low pH (aciduric), and frequently coexists with S. mutans sustaining an acidic biofilm microenvironment (Tanner et al. 2011; Kressirer et al. 2017; Kameda et al. 2020; Damé-Teixeira et al. 2020), dental plaque consists of a polymicrobial community and interactions with other bacteria were not considered. Some formulations contained multiple active ingredients, making it difficult to isolate the exact contribution of each component to bacterial inhibition. Future research should address these limitations by incorporating dynamic models simulating fluctuating pH conditions, carbohydrate challenges, or remineralisation-demineralisation cycles typical of cariogenic environments. Also in vivo studies, longitudinal assessments, and microbiome analyses should be conducted to evaluate the broader impact of these mouthwashes on oral health. Although reductions in bacterial load were observed, direct correlation with clinical caries-risk reduction requires further validation through longitudinal clinical studies.

## Conclusion

The present study provides critical insights into the antimicrobial potential of fluoride-free mouthwashes against *S. wiggsiae*, identifying hydrogen peroxide- and cetylpyridinium chloride-based formulations as the most effective options for reducing bacterial growth, planktonic cell formation, and biofilm development. Essential oil and organic-based formulations exhibited moderate antimicrobial activity, while zinc chloride and stabilised chlorine dioxide formulations showed limited effectiveness against *S. wiggsiae*.

Given the emerging role of *S. wiggsiae* in cariogenic biofilms, particularly in patients with recurrent or severe caries, these findings suggest that certain fluoride-free mouthwashes may serve as valuable adjunctive tools for biofilm control and caries prevention, especially in individuals unable to tolerate or benefit fully from fluoride-based interventions.

## Data Availability

No datasets were generated or analysed during the current study.
